# Genome sequence of *Pseudomonas aeruginosa* bacteriophage

**DOI:** 10.1128/mra.01277-25

**Published:** 2026-01-26

**Authors:** Rafwana Ibrahim, Shaila Angela Lewis, Jesil Mathew Aranjani

**Affiliations:** 1 Department of Pharmaceutical Biotechnology, Manipal College of Pharmaceutical Sciences, Manipal Academy of Higher Education 76808 , Manipal, India; 2 Department of Pharmaceutics, Manipal College of Pharmaceutical Sciences, Manipal Academy of Higher Education 76808 , Manipal, India; Portland State University, Portland, Oregon, USA

**Keywords:** bacteriophage, novel lytic phage, *Pseudomonas aeruginosa*, genome analysis

## Abstract

We describe the isolation of *Pseudomonas* phage SW_PA2862_7_24, a lytic bacteriophage, from sewage water in Manipal, India. It has a double-stranded DNA genome of 92,798 bp in length with a GC content of 49.35%, and the annotated genome shows similarity to *Pseudomonas* phage PJNP029, consistent with its prolate myovirus-like morphology.

## ANNOUNCEMENT

**Fig 1:**
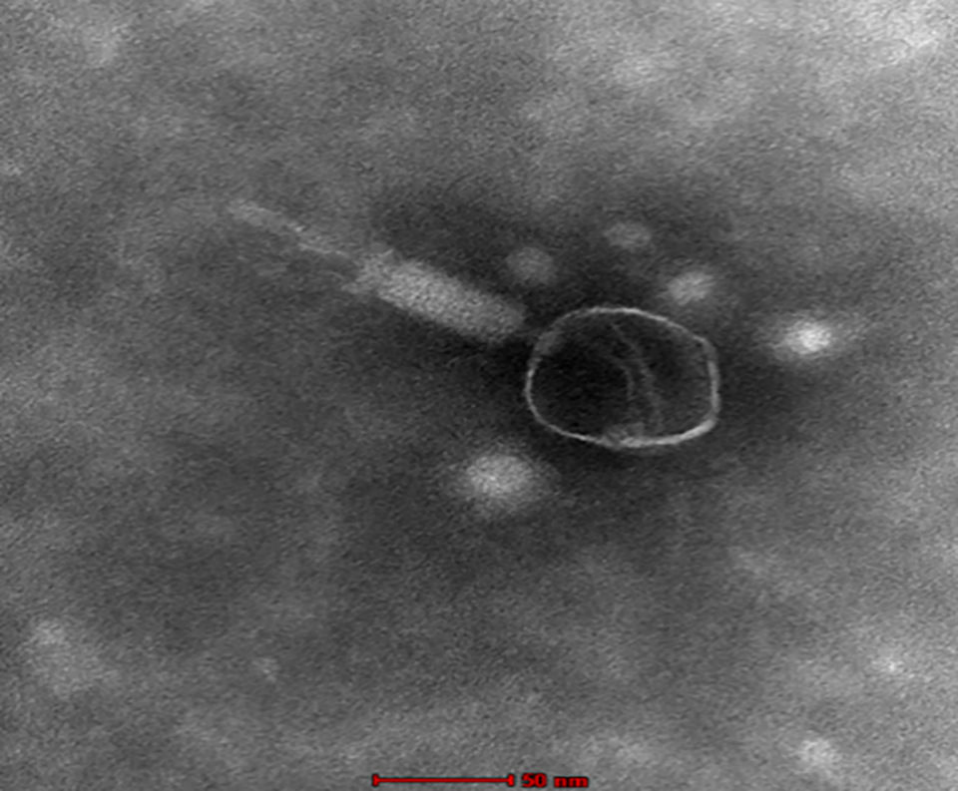
Transmission electron micrograph of the isolated bacteriophage showing an icosahedral head and a contractile tail, characteristic of the Myovirus morphology. The structural features confirmed its classification on the basis of morphological traits. Scale bar: 50 nm.

Bacteriophages, which are viruses that infect and lyse bacteria, are being widely investigated as alternatives to antibiotics for managing drug-resistant bacterial infections (1, 2). Bacteriophage was isolated from a filtered (0.2 μm) sewage water sample collected before treatment at Manipal, India (13.368669, 74.787738), on 12 December 2023. The phage was propagated on *Pseudomonas aeruginosa* 2862 (NCIM) aerobically at 37°C in Luria-Bertani broth (Himedia) using the soft-agar overlay method as described by Akremi et al. (3). After propagation, the phage lysate was clarified by centrifugation (10,000 × *g*, 10 min, 4°C) and filtered through a 0.22 µm membrane to remove bacterial cells. High-quality genomic DNA was isolated from the filtered phage lysate using the QIAamp DNA Mini Kit (Qiagen). DNA quality was assessed by agarose gel electrophoresis and spectrophotometry (A260/280 ratio), and concentration was measured with the Qubit broad range dsDNA quantification kit (Thermo Fisher Scientific). A total of 250 ng of DNA was used for library preparation with the QIASeq FX DNA kit (Qiagen) following the manufacturer’s instructions. The indexed libraries were sequenced on an Illumina NextSeq 2000 platform using 300-cycle paired-end chemistry. Library fragment size was analyzed with an Agilent 4200 TapeStation system, yielding an average insert size of 534 bp. Raw reads were quality controlled using FastQC v0.11.9 and trimmed with fastp v0.12.4 to remove adapter sequences (4, 5). Sequencing generated 1,328,897 paired-end raw reads (2 × 150 bp), resulting in an average genome coverage of approximately 4,300×. Quality assessment of genome assemblies generated by MEGAHIT v1.2.9 (6) was performed using QUAST (7) and CheckV. CheckV classified the final assembled phage genome as complete and high quality and suggested the presence of direct terminal repeats. Genome ends were inferred based on CheckV predictions; no additional termini-detection tools (e.g., PhageTerm) were applied. Assembled genomes were annotated using Pharokka (8), which identifies CDS, tRNAs, tmRNAs, and CRISPRs and provides functional annotation; classification was done using geNomad (https://portal.nersc.gov/genomad/). All software tools were executed using default settings unless otherwise noted. Transmission electron microscopy (TEM) confirmed the myovirus-like morphology of phage SW_PA2862_7_24 (Fig. 1). Imaging was performed using a Tecnai 12 TEM at 80 kV with uranyl acetate staining and Tecnai Imaging Analysis software, following the method of Meidaninikjeh et al. (9).

*Pseudomonas phage SW_PA2862_7_24* was assembled into a single contig of 92,798 bp, with a GC content of 49.35%, and encodes 202 predicted protein-coding sequences, including 15 tRNA genes. Structural annotation identified modules for DNA replication (17 genes: DNA polymerase, primase/helicase, ligase, ribonucleotide reductase, dCMP deaminase), morphogenesis (11 tail proteins, 10 head/DNA packaging genes including large terminase subunit and portal protein), and host lysis (7: endolysins, holins, spanins), indicative of an obligately lytic lifestyle. The largest ORF encodes a 2,058-bp tail fiber protein implicated in host specificity. No lysogeny, virulence, or antibiotic resistance genes were detected. Notably, 63.9% of all ORFs encode hypothetical proteins, reflecting considerable genetic novelty. By BLASTn, SW_PA2862_7_24 shares nucleotide identity with PJNP029 (95.67%, OR941787.1), phipa10 (94.83%, NC_073620.1), and bmx-p3 (97.04%, OQ319933.1), displaying the genomic modularity typical of virulent *Pseudomonas* phages (10, 11).

## Data Availability

The genome sequence of *Pseudomonas phage_SW_PA2862_7_24* is available in GenBank under accession number PV574404. Biosample accession ID SAMN52629495, Bioproject accession number PRJNA1344175, and SRA number SRR35767945.
